# Numerical simulation for impact of implement of reflector and turbulator within the solar system in existence of nanomaterial

**DOI:** 10.1038/s41598-023-37758-x

**Published:** 2023-07-17

**Authors:** M. Sheikholeslami, M. Jafaryar

**Affiliations:** 1 Department of Mechanical Engineering, Babol Noshirvani University of Technology, Babol, Iran; 2 Renewable Energy Systems and Nanofluid Applications in Heat Transfer Laboratory, Babol Noshirvani University of Technology, Babol, Iran

**Keywords:** Mathematics and computing, Nanoscience and technology

## Abstract

Turbulent flow of oil based hybrid nanofluid within an absorber tube of concentrated solar system has been evaluated in this article. To concentrate the solar irradiation, the parabolic plate has been located below the tube and variable heat flux was considered as the boundary condition of the tube. The presence of a turbulator within the circular tube causes secondary flow to increase. Both thermal (S_gen,th_) and frictional (S_gen,f_) components of irreversibility were reported in outputs. As Re increases, the residence time decreases and lower outlet temperature has been achieved. S_gen,th_ decreases about 57.36% with growth of Re while S_gen,f_ increases about 17.44 times. As the number of rows of tapes increases, the value of S_gen,f_ enhances about 69.23% while the value of S_gen,th_ decreases around 3.67%. Increase of pitch ratio causes S_gen,th_ to decrease about 11.25% while frictional component increases around 76.7%.

## Introduction

At its source, the Sun, solar radiation is a powerful energy source with high temperature and exergy, reaching an irradiance of approximately 63 MW/m^2^. While the amount of irradiation reaching the Earth's surface decreases significantly to around 1 kW/m^2^. However, that limitation could be overcome by utilizing concentrated solar units, which convert irradiation into another form of energy, typically thermal, enabling efficient utilization under high solar flux^1^. Thermal energy is generated from irradiation at the focal point of concentrated units. These systems can be categorized based on their focal geometry, including point-focus reflectors such as parabolic dishes, as well as line-focus reflectors such as parabolic-trough collectors (PTCs). PTCs concentrate irradiation onto a focal line and receiver duct has been positioned. With absorbing the reflected irradiation, fluid becomes warmer. PTCs specifically utilize Direct Normal Irradiance (DNI). This refers to the unobstructed irradiation that reaches the Earth’s surface, unaffected by clouds, smoke, or atmospheric particles^2^.

Nanofluids, consisting of solid nanoparticles suspended in base fluids, have gained significant attention as operative heat transfer agents in solar energy usages^3^. Unlike conventional heat transfer fluids, nanofluids exhibit enhanced thermal conductivity, making them promising candidates for improving heat transfer performance. These nanoparticles possess exceptional heat absorption and transport capabilities, making them increasingly popular in solar energy systems. By incorporating nanofluids into solar applications, the thermal features could be considerably enhanced, leading to enhanced efficiency and effectiveness. Nanofluids offer a homogeneous solution for integrating nanoparticles into the base fluid, enabling efficient absorption of solar radiation and facilitating enhanced heat transfer rates on a global scale. Khetib et al.^4^ scrutinized a research on the impact of a disturber in a PTC on the enhancement of performance of a system involving Cu-MgO /H_2_O as working fluid. The findings demonstrated that by increasing the Re, the energy efficiency and exergy efficiency experienced maximum improvements of 23.79% and 21.15%, respectively. A novel twisted strip geometry that enhances the efficiency (η) of solar collectors by reducing (ΔP) was proposed by Ibrahim et al.^5^. Their testing fluid was SWCNT- CuO /H_2_O hybrid nanofluid. Their results indicated that the highest pipe efficiency was achieved when using a strip with three v-shaped cuts, resulting in an efficiency value of 2.46. Influences of loading hybrid nanoparticles on convective flow over a Riga surface have been analyzed by Adnan et al.^6^. They showed that the radiation parameter is the main factor of increment of heat capacity of hybrid nanofluid. Ibrahim et al.^7^ conducted an evaluation to assess the role of a disturber on the enhancement of performance of a solar collector utilizing nanomaterial. They proved that thermal performance enhances about 41.75% with mounting the turbulator and increasing Re leads to augment of exergy performance around 33.09%. To progress the efficiency of PTC, Mustafa et al.^8^ scrutinized a study involving the implementation of twisted tapes through the absorber duct. They employed ANSYS 19.2 software for modeling purposes and demonstrated that augmenting the number of turbulator resulted in improved performance of the solar unit. Notably, they exhibited the greatest efficiency amount of 2.18 under the operating conditions of Re = 25,000 and φ = 2%.

Effect of spring device on turbulent flow within the solar system has been scrutinized by Fuxi et al.^9^. The research findings demonstrated that increasing the size of the disturber led to increased flow turbulence. Yin et al.^10^ studied a comprehensive research on the turbulent flow inside a PTC. Their investigation focused on the utilization of two different spring disturbances. Their output demonstrated that reducing the pitch ratio led to an increase in Nusselt number (Nu). They found that maximum efficiency of 2.39 has been achieved when pitch ratio is 0.44. Numerical investigation was done by Ouabouch et al.^11^ to examine the hydrothermal efficiency of a PTC. They showed that Nu increases with augment of height of the turbulator. Also, they proved that outlet temperature decreases with an increase in the number of turbulator. Influence of radiation and MHD flow on thermal treatment of nanofluid has been scrutinized by Adnan et al.^12^. They analyzed the impact of slip velocity on a vertical surface. In another study, Adnan et al.^13^ analyzed the impact of non-linear radiative heat flux on nanofluid flow through a squeezing duct. Talugeri et al.^14^ conducted an experimental work on a concentrated solar unit. The experiments were carried out between 10:00 and 16:00, with various flow rates. They showed that the final design has 10–11% improvement in performance. Adnan et al.^15^ tried to scrutinize the nanofluid movement over a wedge in existence of radiation. They derived a suitable model based on a similarity approach and utilized experimental data for predicting features of nanofluid.

The previous articles showed that suggesting new configuration of solar system for achieving minimum amount of entropy generation is important. So, present work aims to simulate new configuration of solar collector in existence of parabolic reflector in existence of hybrid nanofluid within the absorber. Complex configuration for disturber has been added within the duct to augment the useful heat and declines the entropy generation. The numbers of revolution and rows of tapes have been considered as geometric parameters and simulations were done for two levels of Reynolds number. Turbulent flow of hybrid nanofluid has been simulated via ANSYS FLUENT and the wall of duct receives non-uniform heat flux which is obtained via SolTrace. Validation test has been done based on previous experimental work. The study of grid assessment leads to best mesh generation procedure. Distribution of temperature and components of irreversibility have been illustrated for various cases. Also quantitative analyze of system in view of irreversibility has been reported.

## The geometry of solar collector and modeling approach

**Figure 1:**
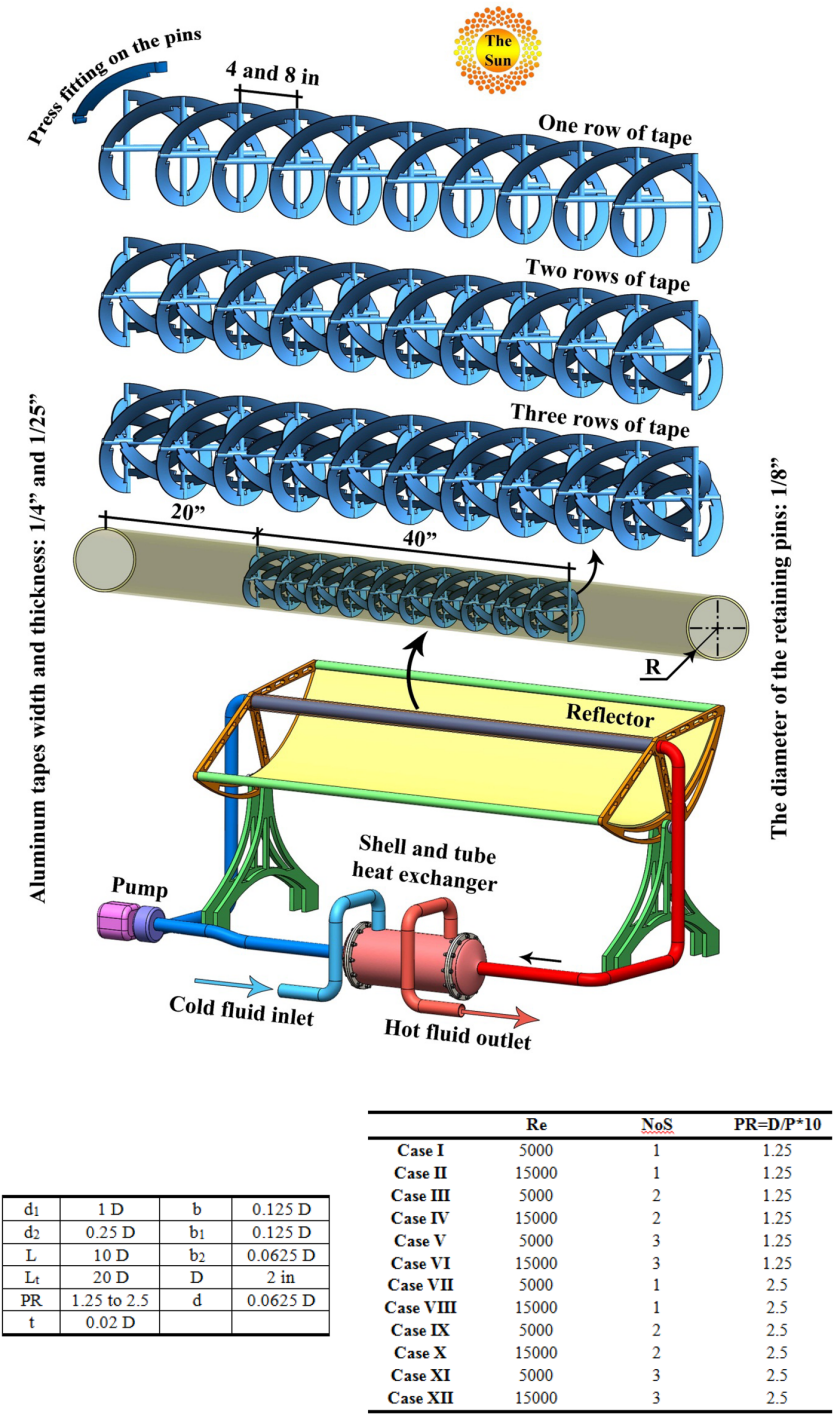
Geometry of concentrating solar system in existence of turbulator.

The parabolic reflector has been installed on the ground and the absorber pipe has been located at the focal distance of the parabolic reflector. As depicted in Fig. 1, a turbulator with three levels of rows have been installed to enhance the swirl flow within the tube. The middle (40 inch) has been assumed as the test section. The dimensions have been shown in this figure. With changing inlet velocity, two levels of Re have been obtained. Also, with changing pitch ratio (PR) and number of rows (NoS), various geometries for the turbulator have been obtained which are simulated numerically. The testing fluid through the duct is a mixture of oil and hybrid nano-powders (MWCNT and Al_2_O_3_) and properties have been derived from Refs.^16,17^. The volume fraction hybrid nanomaterial is 0.01 and temperature dependent properties have been applied. To measure the value of Re, the properties at inlet temperature (293.15 K) have been applied. The details of all twelve cases which were simulated in present work have been declared in Fig. 1. The turbulent flow of working fluid has been simulated according to below equations^18,19^:

1 $$\frac{{\partial \left( {\rho_{hnf} u_{i} } \right)}}{{\partial x_{i} }} = 0$$

2 $$\frac{\partial p}{{\partial x_{i} }} + \rho_{hnf} \left[ { - \frac{\partial }{{\partial x_{j} }}\left( { - \overline{{u_{j}^{\prime } u_{i}^{\prime } }} } \right) + \frac{\partial }{{\partial x_{j} }}\left( {u_{j} u_{i} } \right)} \right] = \frac{\partial }{{\partial x_{j} }}\left( {\left( {\frac{{\partial u_{j} }}{{\partial x_{i} }} + \frac{{\partial u_{i} }}{{\partial x_{j} }}} \right)\mu_{hnf} } \right)$$

3 $$\frac{\partial }{{\partial x_{i} }}\left( {u_{i} \rho_{hnf} T} \right) = \frac{\partial }{{\partial x_{i} }}\left( {\frac{\partial T}{{\partial x_{i} }}\left( {\frac{{\mu_{hnf} }}{{\Pr_{hnf} }} + \frac{{\mu_{t} }}{{\Pr_{t} }}} \right)} \right).$$

The utilized model for turbulent flow has following forms of equations^18^:

4 $$G_{k} - \rho_{hnf} \varepsilon + \frac{\partial }{{\partial x_{j} }}\left( {\frac{\partial K}{{\partial x_{j} }}\left( {\mu_{hnf} + \mu_{t} /\sigma_{k} } \right)} \right) = \frac{\partial }{{\partial x_{i} }}\left( {K\rho_{hnf} u_{i} } \right),\;G_{k} = \rho_{hnf} \left( { - \,\overline{{u_{j}^{\prime } u_{i}^{\prime } }} \frac{{\partial u_{j} }}{{\partial x_{i} }}} \right)$$

5 $$\frac{\partial }{{\partial x_{i} }}\left( {\varepsilon u_{i} \rho_{hnf} } \right) = C_{1\varepsilon } \frac{\varepsilon }{k}G_{k} + \frac{\partial }{{\partial x_{j} }}\left( {\frac{\partial \varepsilon }{{\partial x_{j} }}\left( {\mu_{t} \left( {\sigma_{\varepsilon } } \right)^{ - 1} + \mu_{hnf} } \right)} \right) - C_{2\varepsilon } \rho_{hnf} \frac{{\varepsilon^{2} }}{k}$$

6 $$\sigma_{k} = 1,\;C_{\mu } = 0.0845,\;C_{2\varepsilon } = 1.68,\;C_{1\varepsilon } = 1.42,\;\Pr_{t} = 0.85,\;\sigma_{\varepsilon } = 1.3$$

7 $$\mu_{t} \left( {\frac{{\partial u_{i} }}{{\partial x_{j} }} + \frac{{\partial u_{j} }}{{\partial x_{i} }}} \right) - \delta_{ij} \left( \frac{2}{3} \right)\frac{{\partial u_{k} }}{{\partial x_{k} }}\mu_{t} - \frac{2K}{3}\rho_{hnf} \delta_{ij} = \left( { - \rho_{hnf} \overline{{u_{j}^{\prime } u_{i}^{\prime } }} } \right)$$

8 $$\mu_{t} = C_{\mu } \rho_{hnf} \frac{1}{\varepsilon }K^{2} .$$

**Figure 2:**
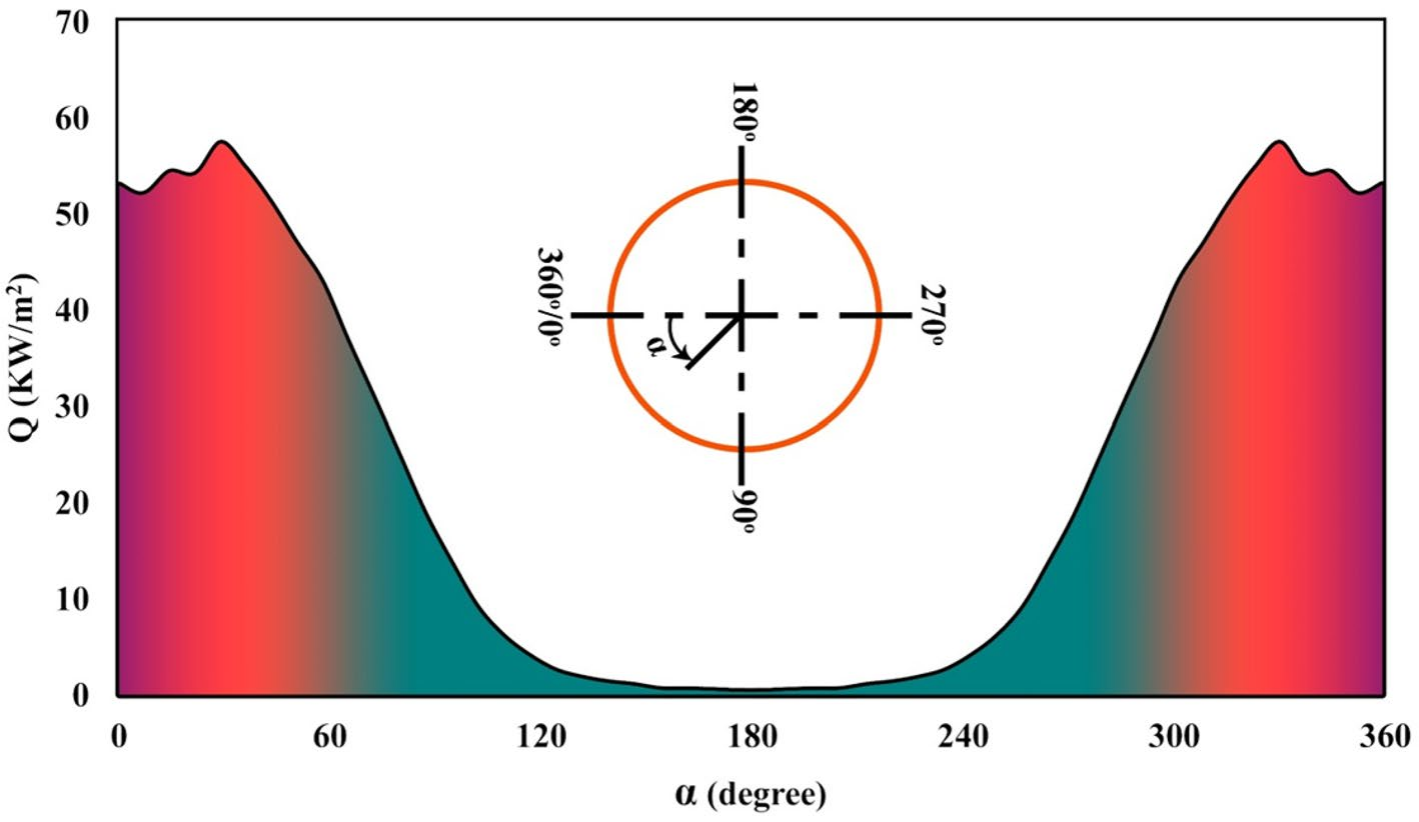
Heat flux received to absorber.

**Figure 3:**
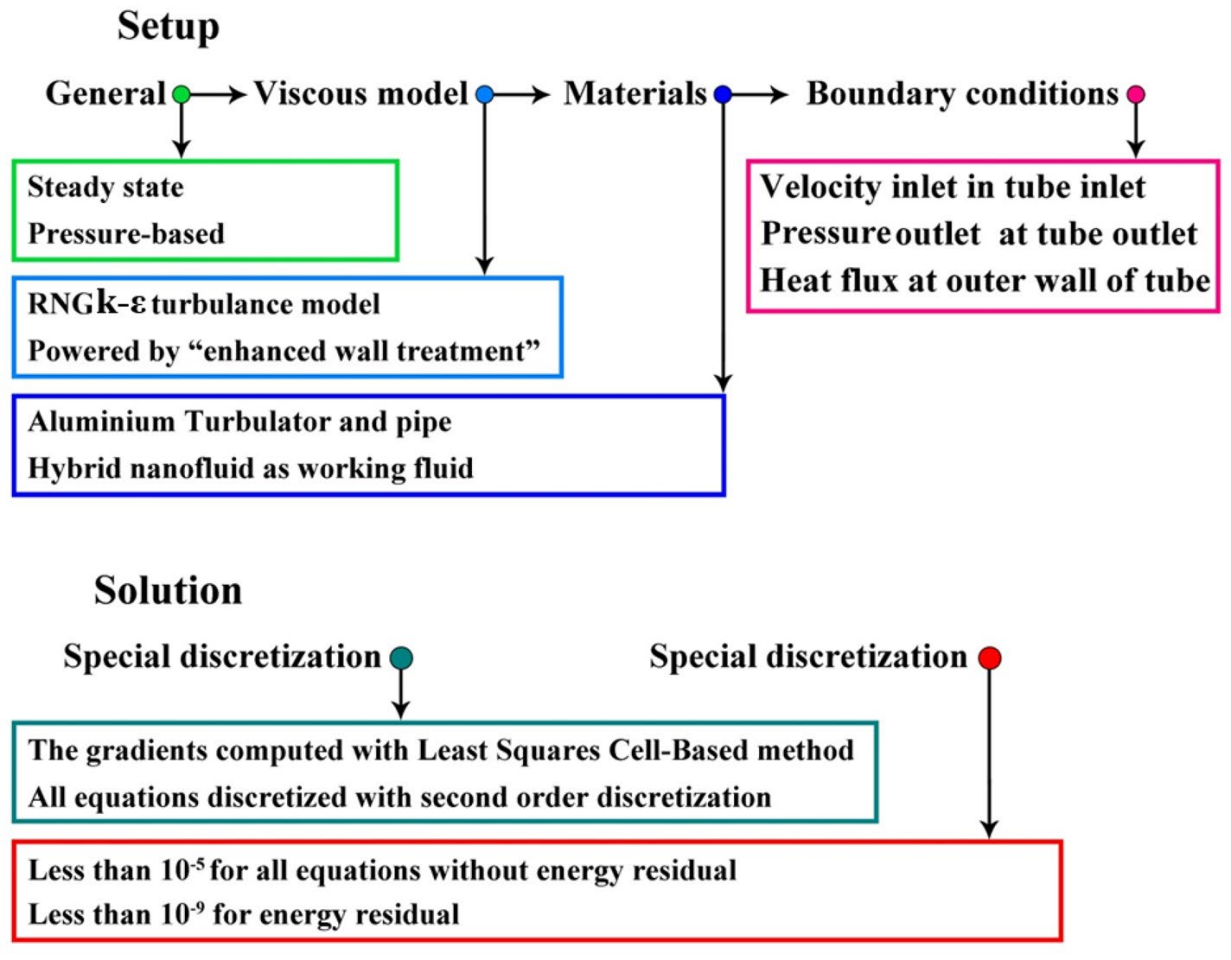
FLUENT set up in simulation.

The rim angle of the reflector is 80°, and its width is 4.52 m. Its obtained concentration ratio is 82. According to SolTrace simulation, the curve of absorbed irradiation has been obtained as revealed in Fig. 2. The details of mentioned software have been mentioned in Ref.^20,21^. In this figure, the angle of 180° denotes the upper point of the tube. For solving the present work, ANSYS FLUENT software has been employed and details of set up have been summarized in Fig. 3. The simulations were done in steady state conditions incorporating pressure based algorithms. Turbulent modeling has been done via k-ε. The solid turbulator and pipe were made from aluminum. The methods for discretization have been mentioned in Fig. 3 and residual for all scalars were less than 10^−5^.

## Results and discussion

To gain more heat flux from the solar system, parabolic reflector has been applied in present work. Concentrated solar systems can absorb high levels of solar irradiation and can be utilized in industrial applications. To increase the thermal output of the solar unit, the absorber can be equipped with a turbulator. For present work, a complicated configuration of a turbulator which is fabricated from various numbers of rows of tapes has been applied. Also, to increase the conductivity of working fluid (oil), hybrid nano-powders have been utilized. Utilizing a homogeneous mixture of additives and oil makes the irreversibility of the system to decrease. 3D turbulent flow within the absorber has been simulated numerically. The impact of presence of reflector has been involved in calculating the received heat flux around the tube. Numbers of rows for tapes (NoS), pitch ratio (PR) and Re have been selected as variables. The entropy generation of the system has been assessed in the result section. Twelve cases were simulated and results in forms of contours and plots were illustrated.

**Figure 4:**
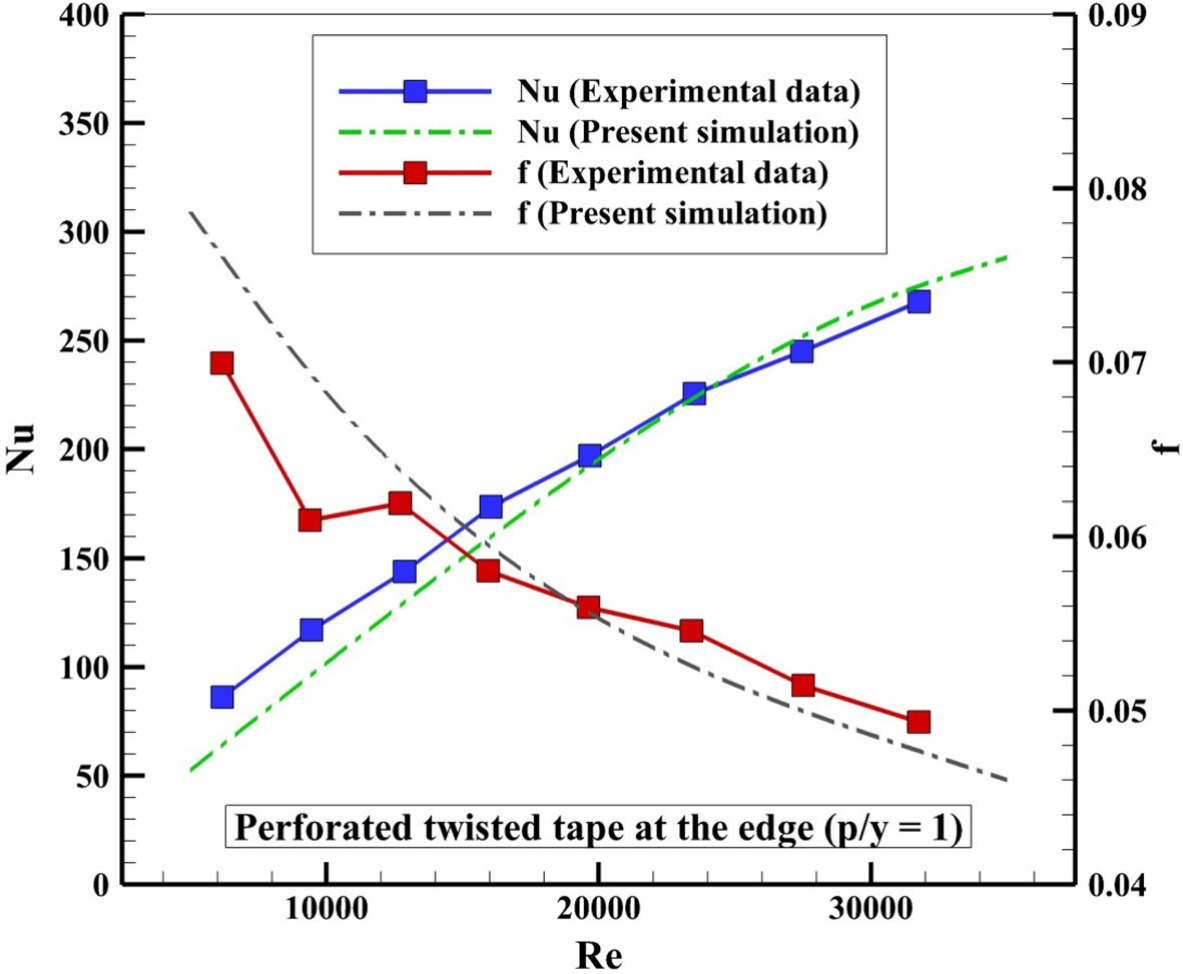
Examination for checking correctness of code against data of Ref.^22^.

Checking the correctness of modeling should be considered as the first step of modeling. The modeling of turbulent flow in the existence of a turbulator is the main important step of present simulation, thus, the selected article for validation is about the experimental evaluation of performance of a tube with dimples and disturber. The previous experimental work^22^ has been considered and amounts of *Nu* and *f* for various ranges of Re were compared (see Fig. 4). Good agreement exists between current outputs and empirical data. Thus, the current code can be employed for the current solar system in existence of a complex disturber.

**Figure 5:**
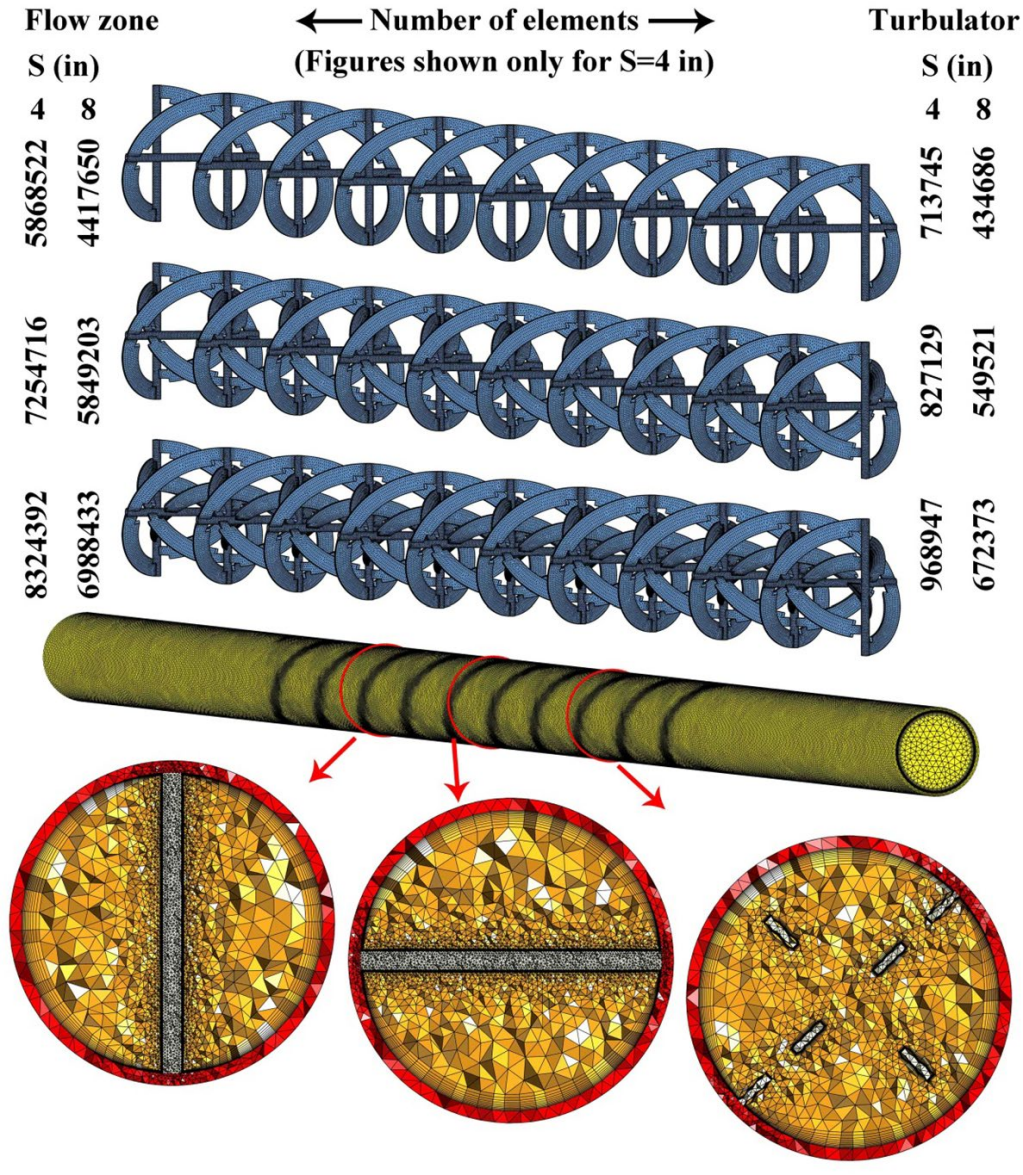
Sample of grid and its details for the most complicated case.

**Figure 6:**
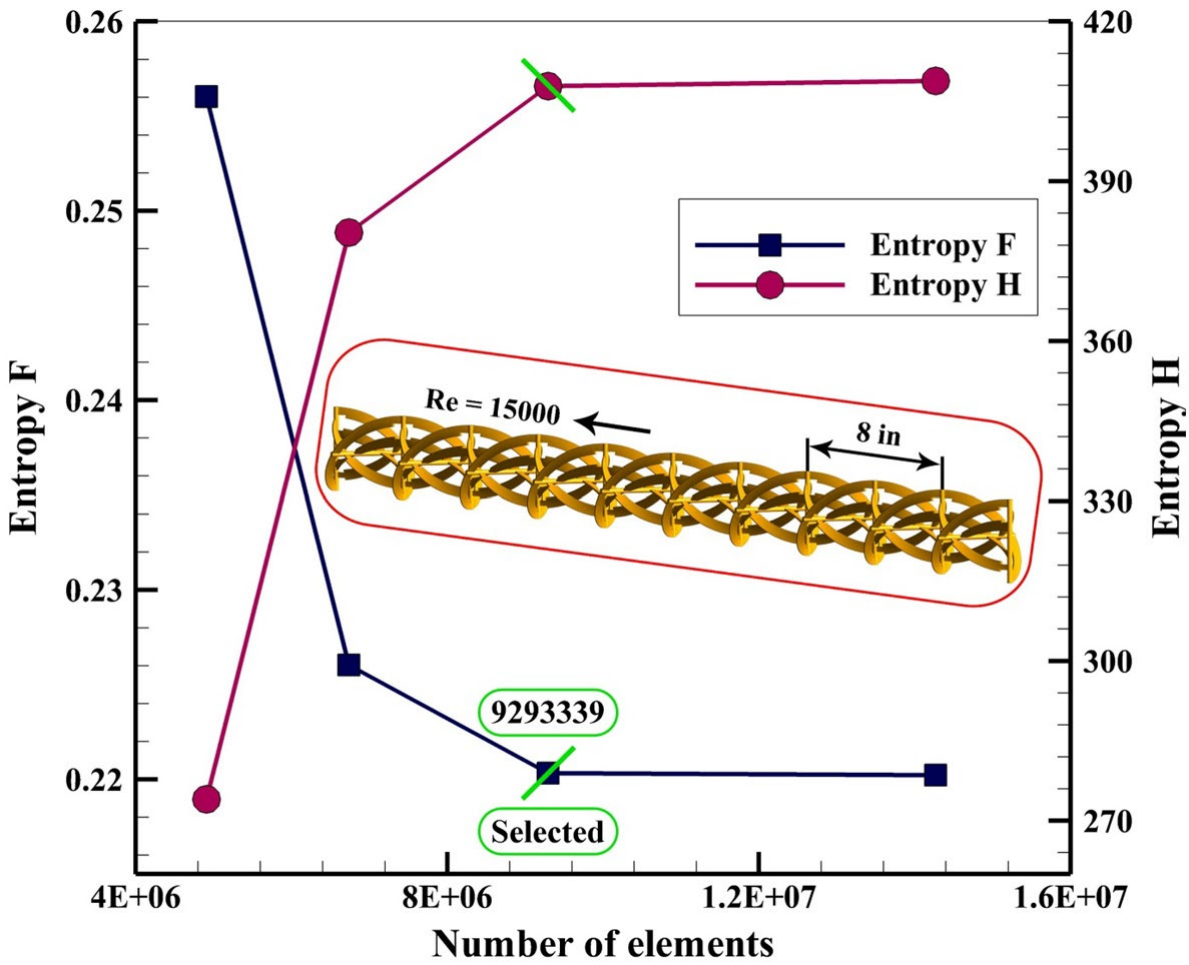
Finding the optimized grid.

The grid analysis is a significant step in modeling of turbulent flow and as demonstrated in Fig. 5, finer grid has been applied near the solid wall to satisfy the acceptable amount of Y^+^. The numbers of elements which were utilized for various cases have been mentioned in this figure and one sample of the lowest level of pitch ratio. To evaluate the various resolutions of the grid, two components of irreversibility have been calculated for each grid. The formulas for calculating these functions are the same as Ref.^21^. Four sizes for the grid have been applied and obtained results for the 12th case (the most complicated case) have been shown in Fig. 6. For this case, the optimized number of total elements is 9,293,339. With selecting such a grid, good accuracy can be obtained involving the acceptable range of Y^+^ for the utilized turbulent model.

**Figure 7:**
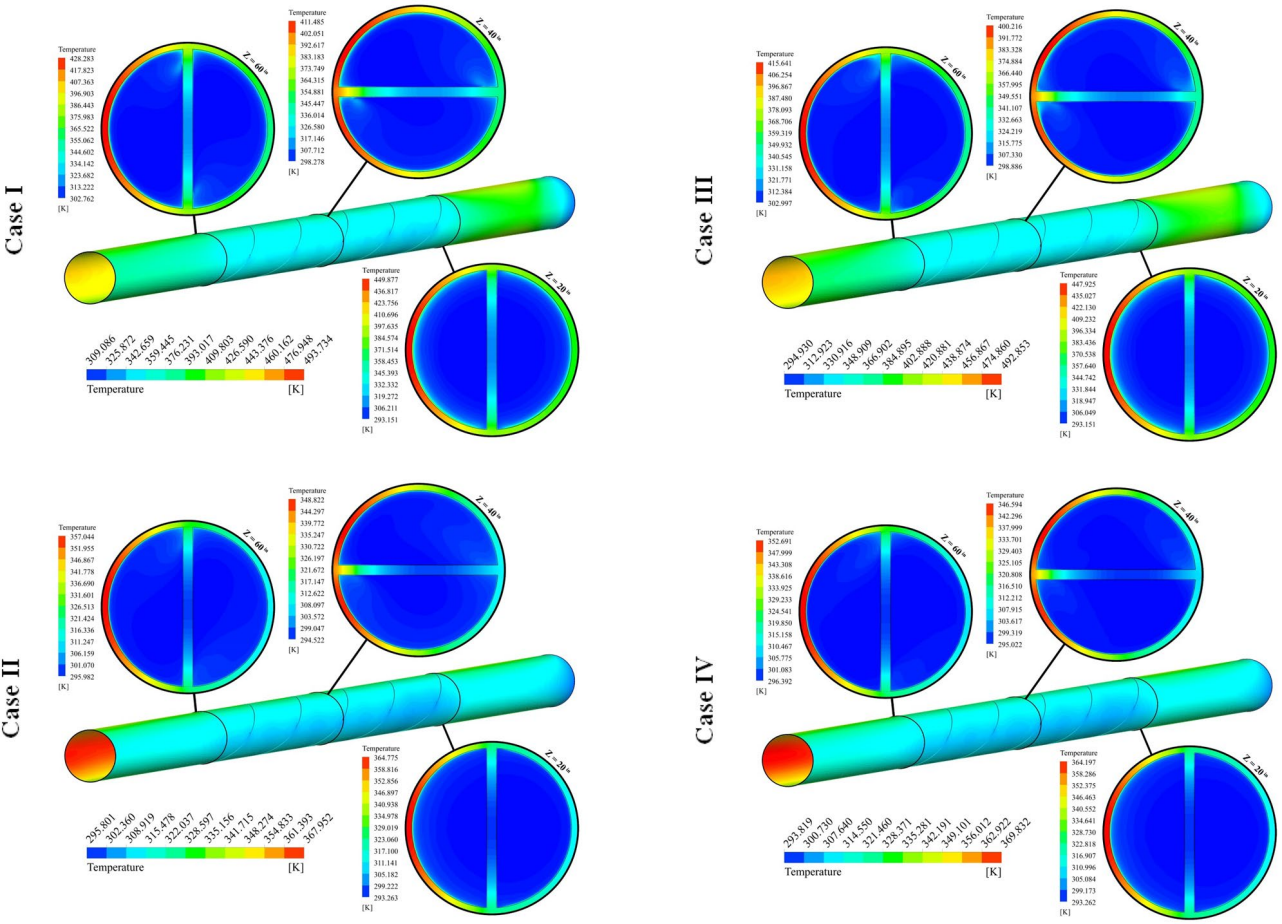

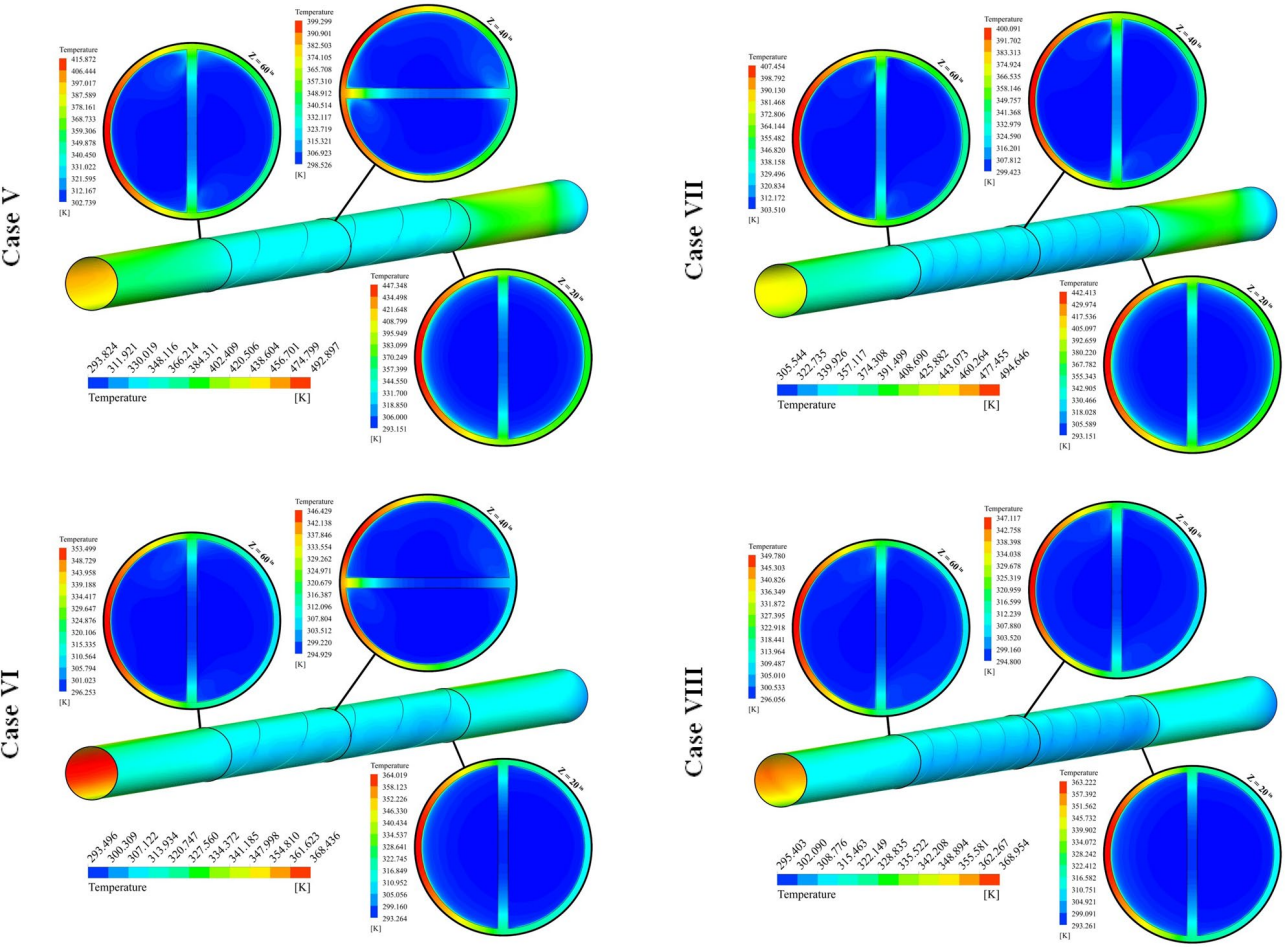

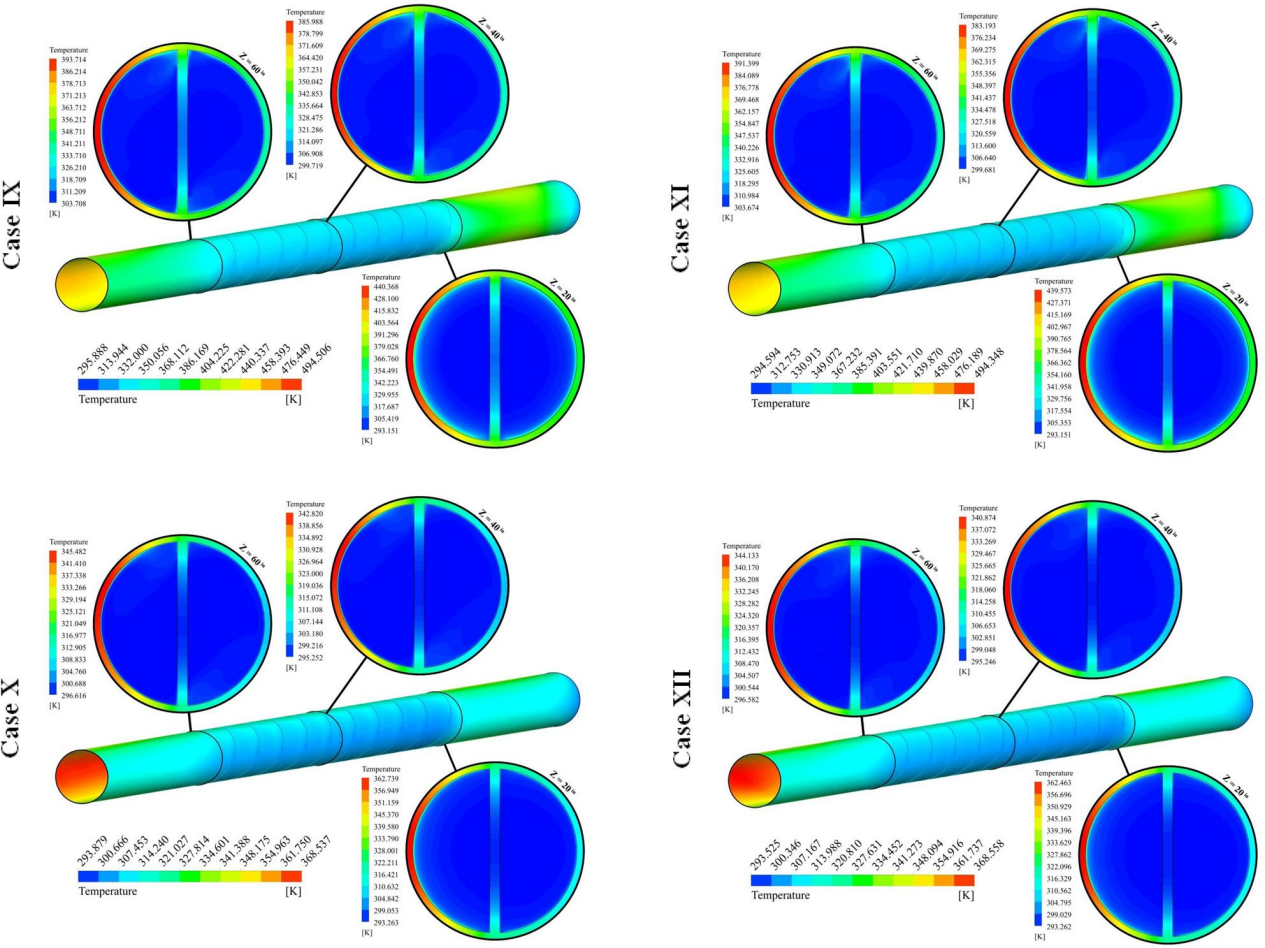
Exhibition of T on various spaces.

The different cases in form of distribution of *T* were illustrated in Fig. 7. In this figure, the temperature over the tube has been demonstrated and isotherms for nanofluid have been shown in three various sections. The region which experiences higher heat flux has higher temperature. With the rise of Z, the temperature of nanofluid decreases. With installing the turbulator within the test section, the temperature of the wall in such a region reduces because of stronger swirl flow in the existence of the turbulator. Utilizing hybrid nanofluid instead of pure oil makes the amount of absorbed heat increase and higher thermal performance must be achieved. With changing the cases, three active parameters have been changed. With the rise of the number of rows, the residence time increases and interaction of hybrid nanofluid with walls increases, thus, temperature of hybrid nanofluid at outlet section enhances. With an augment of pitch ratio, the influence of NoS increases about 2.54% at Re = 5000. Also, the influence of NoS for highest Re is about 5.28 times greater than that of lowest Re. As PR grows, the number of revolutions of the turbulator increases and strength of secondary flow enhances which leads to slightly increment of T_out_. The impact of PR on T_out_ decreases around 88.92% incorporating highest levels of NoS and Re. As pumping power grows, the fluid can move faster within the tube and resident time decreases, thus, temperature of hybrid nanofluid decreases while absorption heat increases because of higher mass flow rate.

**Figure 8:**
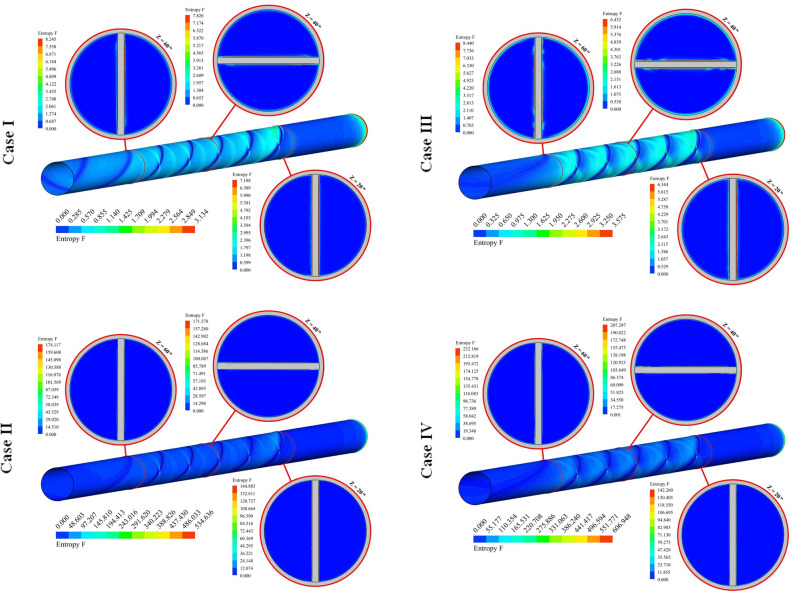

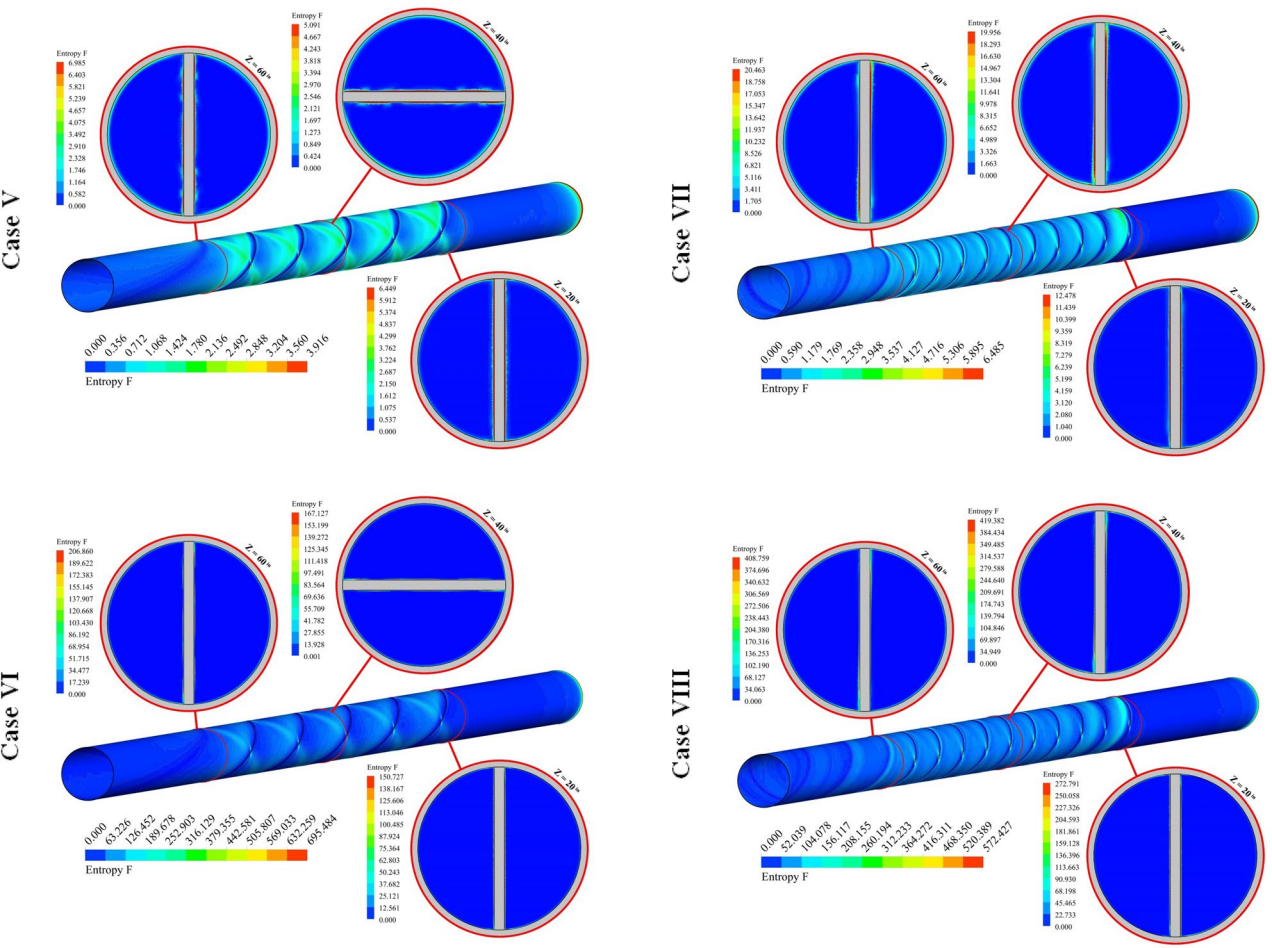

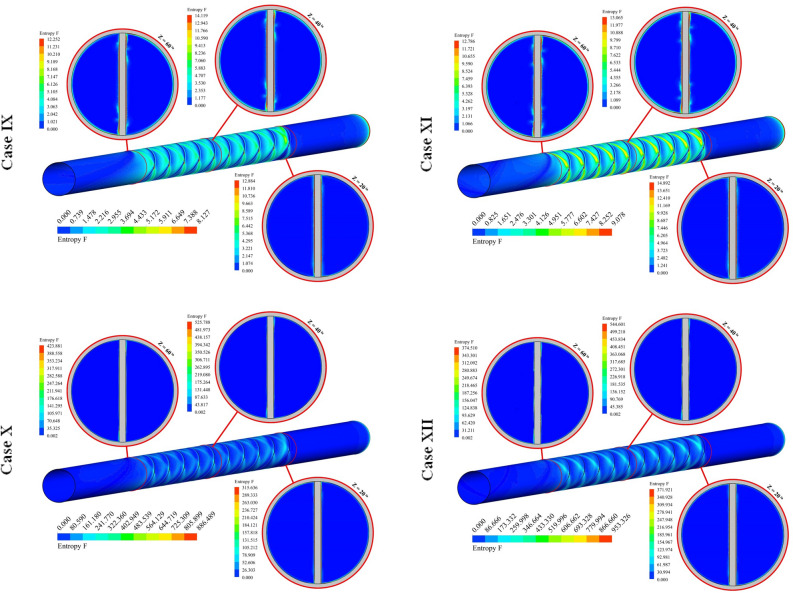
Exhibition of S_gen,f_ at various spaces.

The distributions of S_gen,f_ for 12 cases have been exhibited in Fig. 8. The friction term of irreversibility has a direct relationship with velocity gradient. So, the presence of disturber can increase this function. Augmenting NoS makes the tape configuration becomes more complicated and the strength of swirl flow enhances. As velocity of hybrid nanofluid enhances, the amount of bulk velocity enhances and stronger interaction with the outer wall occurs. So, frictional irreversibility augments with the rise of Re. With increase of revolution of the turbulator, pressure drop increases and velocity gradient increases, thus, S_gen,f_ increases.

**Figure 9:**
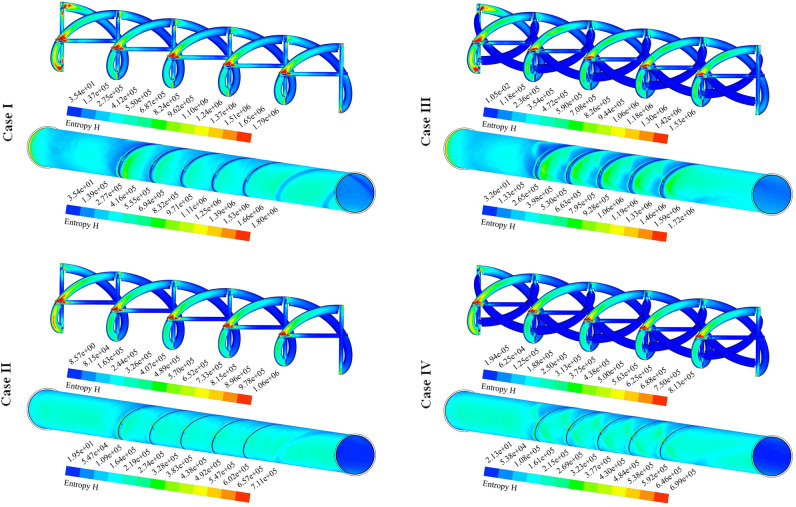

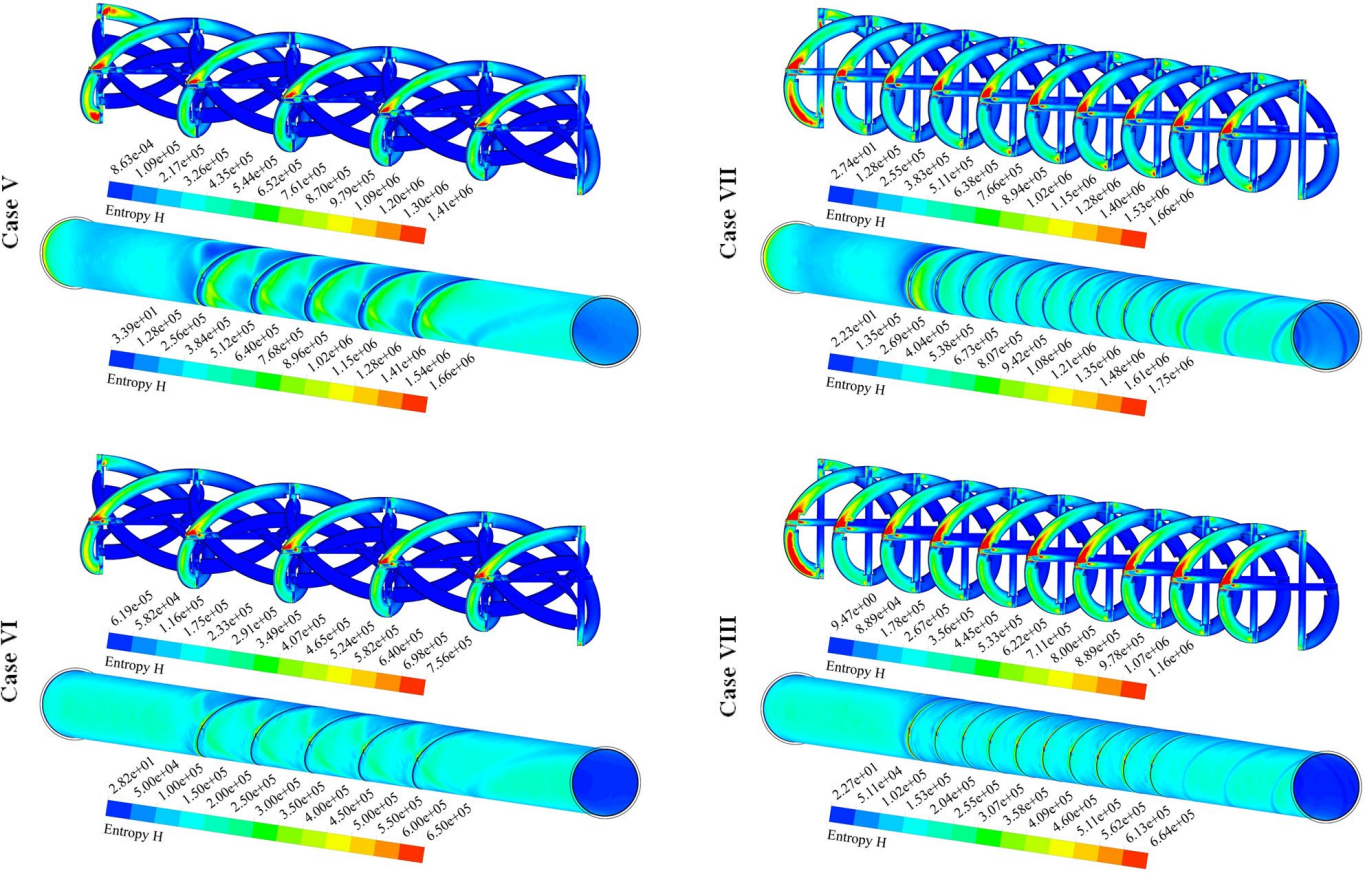

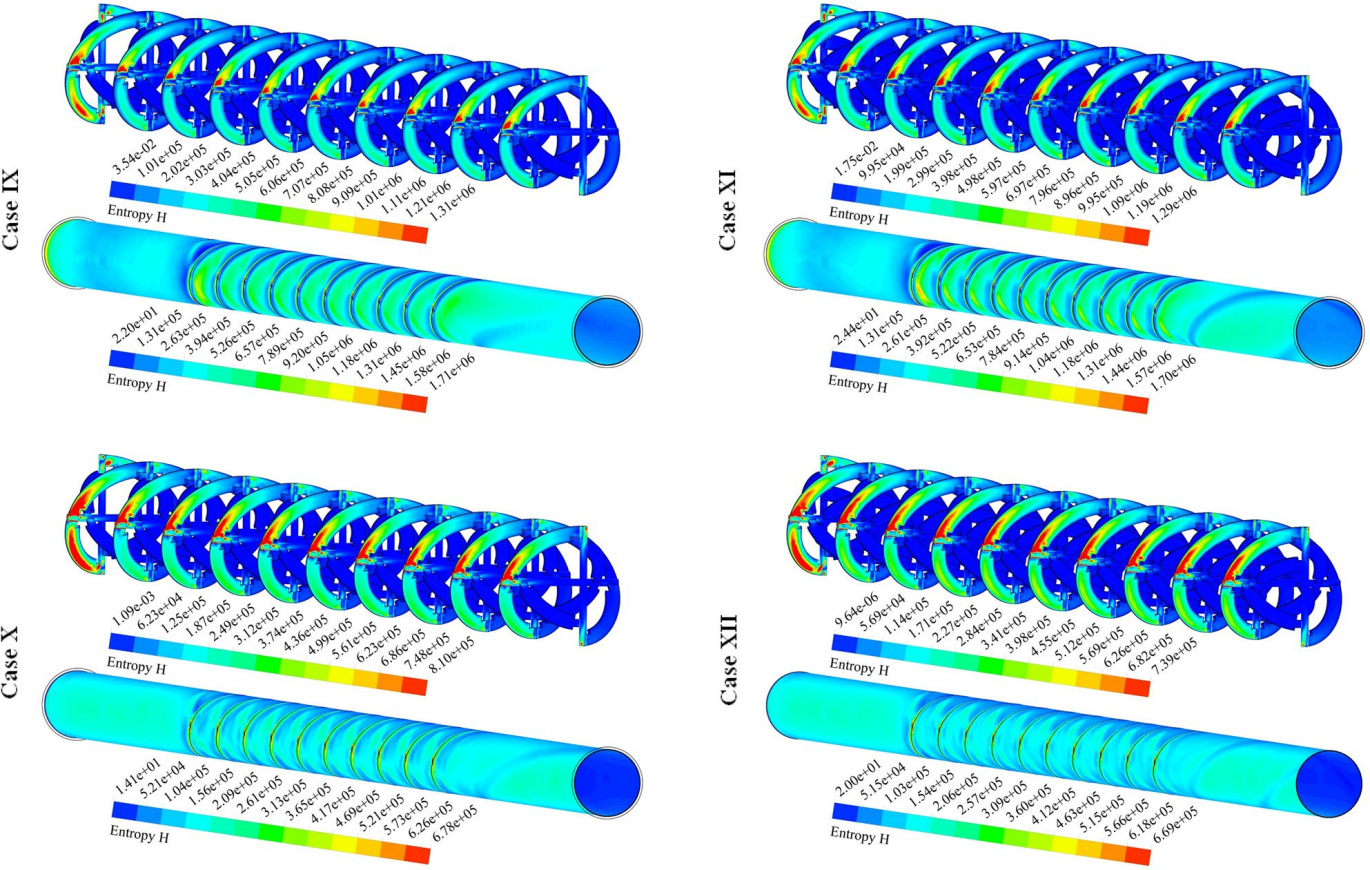
Exhibition of the distribution of S_gen,th_ of hybrid nanofluid.

Figure 9 demonstrates the contour of S_gen,th_ for various cases. The thermal component of entropy generation can be enhanced with rising temperature gradients. Loading hybrid nano-powders can enhance the temperature of fluid and gradient of this scalar reduces, thus, utilizing such testing fluid can reduce the irreversibility of the system. The stronger impingement of hybrid nanofluid with wall occurs if value of PR increases. Therefore, heating irreversibility decreases with the rise of PR. The stronger swirl flow in presence of greater Re leads to decrement of S_gen,th_ owing to reduction of ∆T. The same treatment was illustrated when the number of rows increases while the impact of Re is more sensible in comparison to NoS.

**Figure 10:**
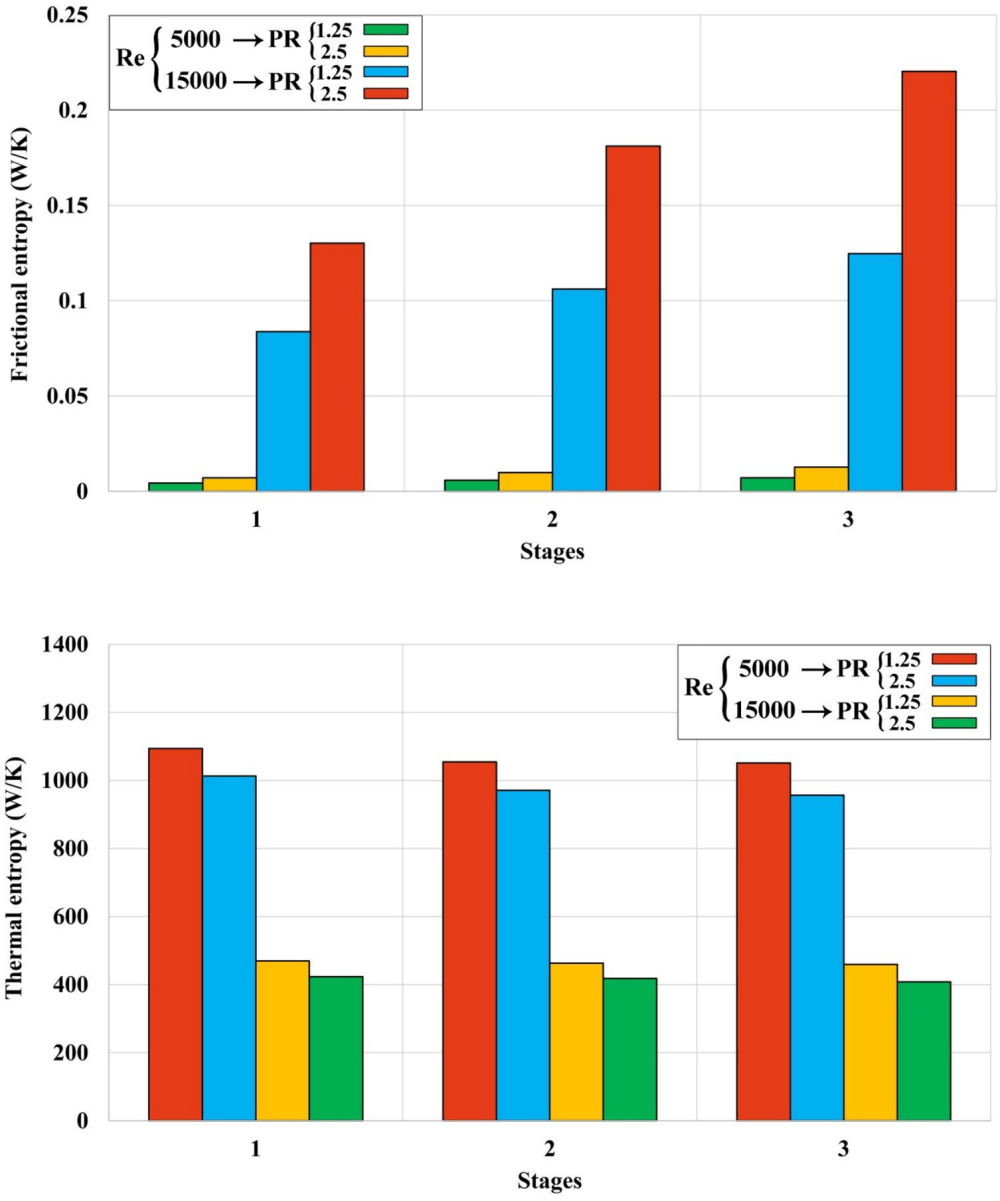
The calculated amounts of *S*_*gen*_ for 12 cases.

The average amounts of S_gen_ were exhibited in Fig. 10. Minimizing the irreversibility of a system should be considered as an important issue in designing a system. By means of simulations, designers can find the regions which have greater irreversibility then find ways to reduce the entropy generation in those regions. Presence of a turbulator with larger values of PR and NoS can reduce the irreversibility of the unit. The lowest and greatest amounts of S_gen_ have been reported for 12th and 1st cases, respectively. As Re grows, the amount of S_gen,th_ decreases about 57.36%. The amount of S_gen,f_ for highest Re is around 17.44 times higher than that of lowest Re. As number of rows has been increased within tapes, the frictional irreversibility augments about 69.23% while S_gen,th_ decreases about 3.67%. The impact of NoS on S_gen,th_ and S_gen,f_ increased about 76.55% and 41.27% in presence of greater value of PR. Also, the influence of NoS can be more sensible when lower pumping power has been applied. With increase of PR, S_gen,th_ decreases about 11.25% while S_gen,f_ increases around 76.7%.

## Conclusion

To increase the thermal output of the solar unit, a reflector with the shape of parabolic has been mounted on the ground. The absorber has been located in the focal line of the reflector to gain the highest solar irradiation. The testing fluid within the absorber is oil which is mixed with hybrid nano-powders. The swirl flow within the tube enhances with installing the turbulator inside the absorber. The turbulator consists of various helical tapes. The number of rows (NoS) and pitch distance (PR) are two geometrical variables of present work. Also, various values of inlet velocity provide various Re which are in turbulent regime. The reasons for adding nano-powders are achieving higher conductivity and lower irreversibility. The components of S_gen_ have been presented for various twelve cases. The simulation for 3D domain in steady state conduction has been done via the Finite volume method. The space dependent heat flux around the absorber has been considered. For calculating the properties of hybrid nanomaterial, single phase formulation has been utilized. The accuracy of present modeling was tested with comparing the outputs with previous empirical data and good accommodation has been applied. The selected previous work is about fluid flow within a tube with dimples including twisted tape, so, such validation shows that the current model has reasonable accuracy of predicting experiment set up. The number of elements in mesh generation should be minimized to reduce computational cost. So, various grids have been applied for different geometry and obtained results for 12th case shows that 9,293,339 elements should be employed to reach the correct modeling. Also, the distance of the first layer from the solid layer of the tube should be selected according to the acceptable range of Y^+^ for RNG k–ε model. Dispersing additives within oil can enhance the heat absorption of working fluid and thermal irreversibility decreases. So, such selection of testing fluid has a beneficiary in both view of energy and entropy generation. With increase of number of rows and revolution of tapes, the turbulator shape becomes more complex and secondary flow increases and hybrid nanofluid can absorb more heat. So, S_gen,th_ decreases with rise of NoS and PR while S_gen,f_ increases with augment of such parameters. Also, temperature of working fluid enhances slightly with increase of mentioned parameters. As velocity of hybrid nanofluid at inlet increases, the fluid bulk velocity increases but outlet temperature decreases. The growth of Re leads to augment of S_gen,f_ while S_gen,th_ decreases around 57.36%. As PR increases, S_gen,th_ reduces around11.25% but S_gen,f_ grows about 76.7%. The amount of S_gen,f_ augments about 69.23% with growth of PR but S_gen,th_ reduces around 3.67%. The influences of number of rows on S_gen,th_ and S_gen,f_ augments about 76.55% and 41.27% in the existence of higher levels of PR. The lowest amount of entropy generation occurs for 12th case.

## Data Availability

All outputs studied during current work are included in this published paper.
